# A New Species and New Synonym in *Heptagenia* Walsh (Ephemeroptera: Heptageniidae: Heptageniinae) Based on Molecular and Morphological Evidence.

**DOI:** 10.1673/031.007.6301

**Published:** 2007-12-21

**Authors:** J. M. Webb, L. Sun, W. P. McCafferty, V. R. Ferris

**Affiliations:** ^1^ Department of Environmental Management and Ecology, La Trobe University, Albury-Wodonga Campus, PO Box 821, Wodonga, Victoria, Australia 3689; ^2^ Department of Entomology, Purdue University, West Lafayette, IN, 47907-2089 USA

**Keywords:** DNA barcode, *Heptagenia whitingi*, new species, new synonym, mayfly, COI

## Abstract

A new mayfly species, *Heptagenia whitingi* Webb & McCafferty n.sp. is described from larvae, a male subimago, a female adult, and eggs collected from large rivers in the west-central portion of North America. Larvae are differentiated from other North American *Heptagenia* Walsh by a pair of large, rectangular pale markings on abdominal tergum 4, and the combination of having the posterior margin of the abdominal terga with bluntly pointed spines less than half the length of the fine setae, small blunt spines on the posterior margin of the caudal filaments, and numerous rows of setae laterally on the ventral surface of the labrum. A 630 bp partial sequence of the mitochondrial gene cytochrome oxidase 1 (COI) from three specimens of *H. whitingi* n.sp. was compared with those of 12 specimens representing eight other North American species of *Heptagenia*. Intraspecific sequence divergences based on Kimura-2-parameter (K2P) distance ranged from 0–1.1%. Interspecific sequence divergence based on K2P distance ranged from 8.9–20.0%. *Heptagenia whitingi* n.sp. differed from its sister taxon *H. flavescens* (Walsh) by 11.7%. *Heptagenia diabasia* Burks and *H. elegantula* (Eaton) differed from each other by only 1.1%; these two alleged species show a clinal pattern in larval abdominal coloration and there are no structural differences between the semaphorants. On this basis, *H. diabasia* is placed as a junior subjective synonym of *H. elegantula*, n.syn.

## Introduction

Mayflies of the genus *Heptagenia* Walsh (Heptageniidae: Heptageniinae) are distributed throughout the Holarctic biogeographic realm. Species reported as *Heptagenia* from Southeast Asia are actually members of other genera (Wang and McCafferty 2004, Webb et al. 2006). In North America, 12 species have been recently recognized, although some of these may prove to be synonymous. No analytical hypothesis of the relationships among the North American species of *Heptagenia* has been proposed. Nonetheless, based on characters of the genitalia the species have been divided into a *flavescens* group that includes *H. flavescens* (Walsh), *H. townesi* Traver, *H. marginalis* Banks, *H. culacantha* Evans, Botts & Flowers, *H. dolosa* Traver, and *H. patoka* Burks; an *elegantula* group that includes *H. elegantula* (Eaton), *H. adaequata* McDunnough, and *H. diabasia* Burks; and *apulla* group that includes *H. pulla* (Clemens), *H. julia* Traver, and *H. solitaria* (McDunnough) (e.g., Traver 1935). Similar species groups have been proposed for the Palearctic species (Kluge 1987).

Male adults of the North American species can generally be identified using keys provided by Traver (1935) and Burks (1953), using a combination of color patterns and genitalic morphology. Females, however, cannot currently be identified to species with any reliability. Keys available for identifying larvae (e.g., Traver 1935; Burks 1953; Bednarik and Edmunds 1980; Webb et al. 2002) are inadequate as they rely almost entirely on color patterns and are mostly regional in scope. Complicating diagnostic issues are data that show that different environmental conditions cause variations in color patterns in heptageniid mayflies (i.e. McCafferty and Pereira 1984) and the fact that color patterns often are not apparent in preserved material. Moreover, the larvae of several species remain unknown.

We have examined specimens of a new species of *Heptagenia* from large rivers in the grasslands and parklands of north-central North America. Specimens of this species had previously been identified as *H. flavescens* (Lehmkuhl 1976; Whiting 1985; Whiting and Sheard 1990; Webb 2002; Webb et al. 2002; Ball et al. 2005). The new species differs from *H. flavescens* in abdominal color pattern and slight morphological differences in the relative size of abdominal spines and setation.

Because the morphological characterization of the new species may appear minimal, we sought to confirm its distinctiveness from other species by employing DNA barcoding. Barcoding has recently been proposed as a possible solution to some of the problems of traditional species identification (Hebert et al. 2003a). A short section, approximately 650 base pairs (bp), of the mitochondrial gene cytochrome oxidase I (COI) is used as a marker for species identification and/or discrimination in barcoding. Species identifications are obtained by comparing unknown sequences with those contained in a reference library of sequences obtained from identified specimens. The ability to successfully identify or discriminate species depends on the presence of a barcoding gap, a distinct difference between intra- and interspecific sequence divergences; in most taxa there is an order of magnitude difference between the two (Hebert et al. 2004; Ball et al. 2005). This method has been applied successfully in many invertebrate taxa, including mayflies (Hebert et al. 2003a,b; Hogg and Hebert 2004; Ball et al. 2005; Monaghan et al. 2005; Hajibabaei et al. 2006).

This study had three objectives: (1) describe a new species of *Heptagenia*; (2) provide barcode data for the new species using a partial sequence of COI; (3) provide preliminary data on the relationships of North American *Heptagenia* species based on COI.

## Taxonomy

### *Heptagenia whitingi* Webb & McCafferty, new species

#### Mature larva (in alcohol)

Body length: 9.5–11 mm.

### Head

Brown with pale markings. Median pale spot near anterior margin usually quadrate in shape, sometimes connected by pale line to pale spot anterior to median ocellus. Lateral pale streaks wide at lateral margin, usually 0.25–0.5 X length of head capsule. Labrum 4X wider than long; ventral surface with single row of robust setae medially and many fine setae laterally (Figure 3). Maxilla with 7–8 comb setae on apical margin; galealacinia ventrally with row of fimbriate setae, dorsolaterally with dense row of fine setae; maxillary palp with many long setae on first segment and with row of setae on second and third segments.

### Thorax

Notum brown with pale markings. Fore femur pale with two brown transverse markings on anterior surface; hind margin with row of long fine setae and long robust setae; anterior surface with paddle shaped setae and fine setae. Fore tibia pale with median brown stripe, surface with scattered short fine setae, row of long fine setae near lateral ridge; lateral ridge with short paddle shaped setae; posterior ridge without row of long fine setae. Fore tarsus pale with brown stripe basally, with numerous scattered fine and paddle shaped setae. Middle and hind legs similar to fore leg except with more numerous setae and tibiae with row of long fine setae on posterior ridge. Claws without denticles.

**Figure 1.**
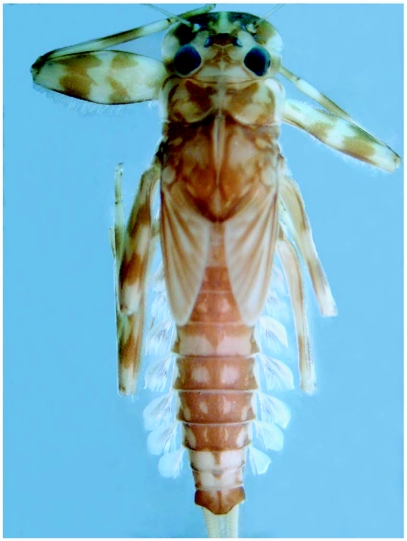
*Heptagenia whitingi* Webb & McCafferty n.sp., dorsal habitus of holotype larva.

### Abdomen

**Figure 2.**
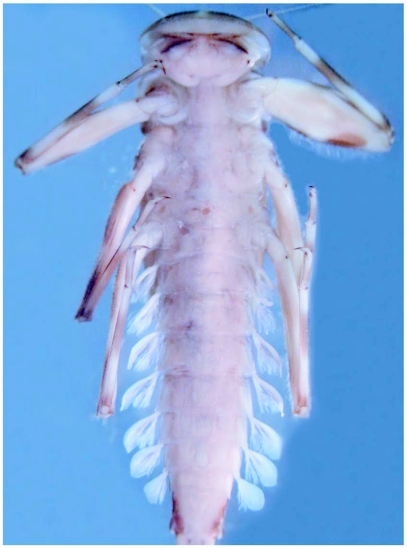
*Heptagenia whitingi* Webb & McCafferty n.sp., ventral habitus of holotype larva.

Terga brown with distinct pale markings (Figure 1) and with numerous fine setae; terga 3–7 with paired pale markings medially, those on tergum 4 large and rectangular in shape; posterior margins with bluntly pointed spines and fine setae, spines less than 1/2 length of fine setae (Figure 4). Sterna mostly pale, sternum 9 brown laterally (Figure 2). Caudal filaments with fine setae; posterior margin of each segment with spines approximately 1/6 length of segment (Figure 5) and sparse robust setae.

### Male subimago (in alcohol, faded and damaged)

Wing length: 11 mm.

Body generally yellowish. Wings hyaline with veins yellowish-brown; interspace of C and Sc of fore wing basally with few, poorly developed cross veins. Legs yellowish. Abdomen brownish dorsally, with pair of pale median marks. Penes with well-developed dorsolateral projections, dorsal protrusion present on each side of midline and with sharp spine; titillators robust. Caudal filaments yellowish.

**Figure 3.**
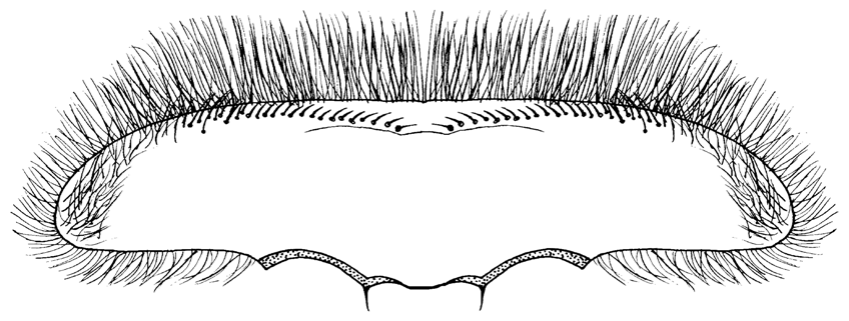
*Heptagenia whitingi* Webb & McCafferty n.sp., ventral surface of labrum showing single median row of robust setae and numerous rows of fine setae laterally.

**Figure 4.**
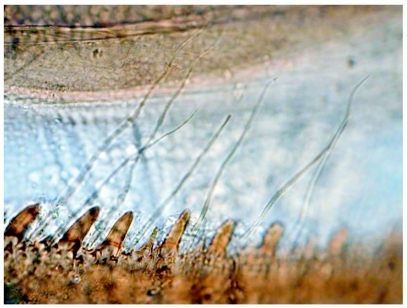
*Heptagenia whitingi* Webb & McCafferty n.sp., posteromedian margin of abdominal tergum 6, showing relative sizes of spines and fine setae.

### Female adult (in alcohol)

Body length 8.5 mm.

Wing length: 10.3 mm.

Body yellowish, slightly darker dorsally. Wings hyaline with longitudinal and cross veins yellowish-brown; interspace of C and Sc of fore wing basally with few, poorly developed cross veins (Figure 6). Legs yellowish. Abdomen pale yellowish, terga 2–7 brown posteromedially. Subgenital plate truncate, posterior margin brown (Figure 7). Caudal filaments pale, slightly darker basally.

**Figure 5.**
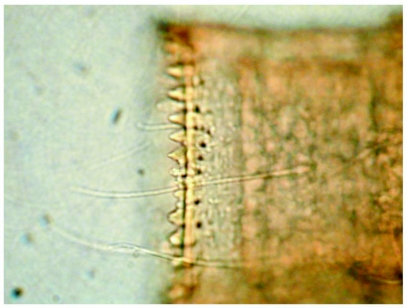
*Heptagenia whitingi* Webb & McCafferty n.sp., spines on poster margin of one segment of the median caudal filament.

**Figure 6.**
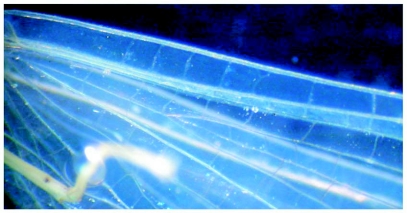
*Heptagenia whitingi* Webb & McCafferty n.sp., female adult, base of fore wing, showing poorly developed cross veins between C and Sc.

**Figure 7.**
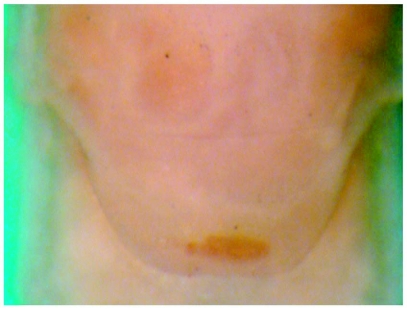
*Heptagenia whitingi* Webb & McCafferty n.sp., female adult, subgenital plate.

**Figure 8.**
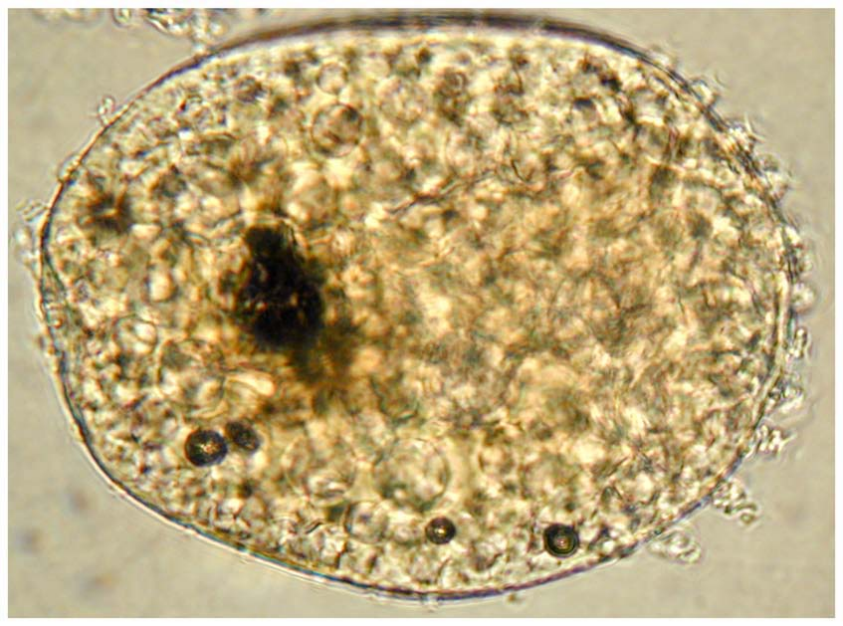
*Heptagenia whitingi* Webb & McCafferty n.sp., egg.

**Figure 9.**
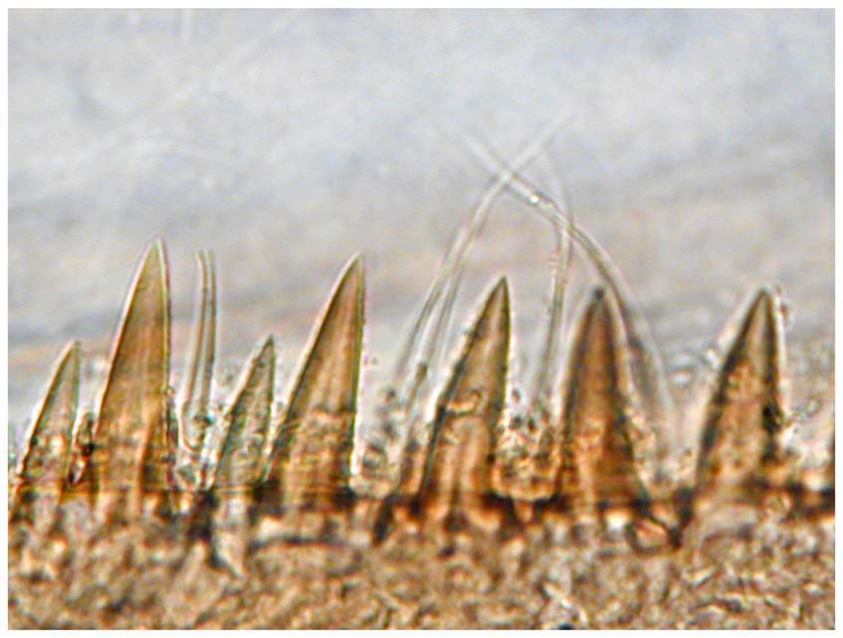
*Heptagenia flavescens*, posterior margin of tergum 6.

### Egg

Length: 159 µm

Width: 98 µm

Chorion covered with numerous knob-terminated coiled threads (KCTs), those at one pole larger and more numerous (Figure 8).

### Material examined

Holotype (in alcohol): Late instar larva, USA, Montana, Blaine Co.: Milk River, 16 miles west of Havre, at U.S. Hwy 2. 48.5958°–109.3633°, 14-VI-2000, W.P. McCafferty et al., deposited in the Purdue Entomological Research Collection, West Lafayette, IN (PERC). Paratypes: 1 larva, same data and deposition as holotype; Canada, Saskatchewan, North Saskatchewan River at Cecil Ferry, 53.2290°–105.5114°: 2 larvae (parts of one slide mounted in Canada Balsam), 15-VI-1986 E.R. Whiting, #JMW713 (PERC); 1 male subimago with associated exuviae, 15-VI-1986 E.R. Whiting, #JMW1914 (PERC); 8 larvae (parts of 1 slide mounted in Euparal), 6-VI-2000, J.M. Webb, #JMW1004 (PERC); 1 female adult with associated exuviae, 28-VIII-2000, J.M. Webb; 3 larvae, 28-VIII-2000, J.M. Webb (PERC); North Saskatchewan River at Borden Bridge, 52.3714°–107.1453°, 2 larvae, 3-V-2000, J.M. Webb, #JMW736 (PERC); South Saskatchewan River at Lemsford Ferry, 51.0300°–109.1200°: 3 young larvae, 23-V-1998, J.M. Webb (PERC); 6 young larvae, 30-VII-2000, J.M. Webb, #JMW1090 (PERC); 8 larvae, 16-IX-2000, J.M. Webb, #JMW1233 (PERC); 7 larvae, 3-VII-2001 J.M. Webb (PERC); 2 larvae, 17-V-2000 J.M. Webb, #JMW1190 (PERC); 7 larvae, 16-X-1999, J.M. Webb (PERC); 13 larvae, 30-IV-1998, J.M. Webb, #JMW1493 (PERC); 1 larva, 3-VII-2001, J.M. Webb, #JMW1616 (PERC). USA: Kansas, Wyandotte Co.: Kansas City, Missouri River at 7th St. Bridge, 39.1562 °–94.6232°, 5 larvae (parts of one on slide), 9-X-1979, KDHE and J. Fry (Snow Entomological Collection, Lawrence, KS); Missouri, Platte Co.: Kansas City, Missouri River at 7th St. Bridge, 39.1562 °–94.6232°,1 larva, 9-X-1979, KDHE and J. Fry (Snow Entomological Collection, Lawrence, KS); Montana, Dawson Co.: Yellowstone River at Intake, 47.2801°–104.5298°, 2 larvae, 17-XII-1974, R.L. Newell (PERC).

### Etymology

The specific epithet is in honor of the late Eric Whiting who collected and studied the Heptageniidae of central Canada.

**Table 1.**
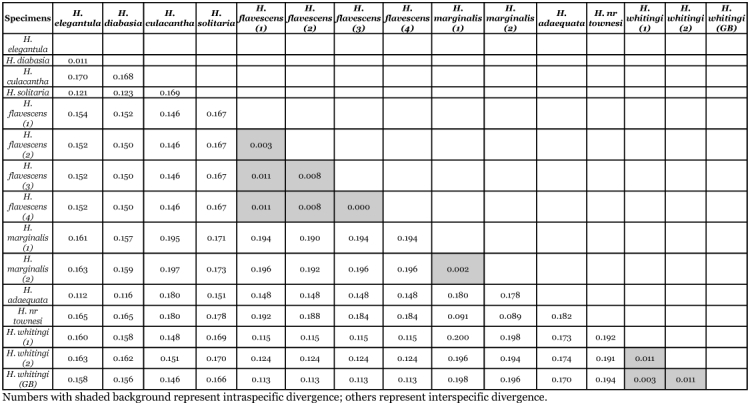
K2P distance values among COI MtDNA partial sequences of 15 North American *Heptagenia* samples. Numbers with shaded background represent intraspecific divergence; others represent interspecific divergence.

## Diagnosis

### Larvae

*Heptagenia whitingi* is differentiated from all other North American congeners by the pair of large, rectangular pale marks on abdominal tergum 4 (Figure 1). Larvae of the new species are structurally most similar to those of *H. flavescens*, but in *H. flavescens*, the spines on the posterior margin of the abdominal terga are sharply pointed and greater than 1/2 the length of the fine setae (Figure 9). The color pattern of some individuals of *H. whiting* and *H. adaequata* can be similar, but the rounded pale median spot on the head capsule of *H. adaequata* should differentiate the two; additionally, the whorl of spines on the caudal filaments of *H. whitingi* are much shorter than those of *H. adaequata* and the tibiae of *H. adaequata* lack a median brown band.

### Adults

It is not generally possible at this time to identify species of *Heptagenia* based on subimagos or females and as such diagnostic characters for the adult stages of *H. whitingi* cannot be provided. The posterior margin of the subgenital plate of female *H. flavescens*, however, is usually either pale or slightly shaded with brown along its entire length, whereas in the female of *H. whitingi*, the posterior margin has a distinct brown marking located medially (Figure 7). The lack of dark longitudinal marks on the abdominal terga of the adults distinguishes adults of *H. whitingi* from those of all other North American *Heptagenia* of the *flavescens* group except *H. flavescens* and *H. patoka*.

### Eggs

The eggs *H. whitingi* do not appear to differ significantly from North American congeners. Several species of North American *Heptagenia* have been previously described (Smith 1935; Koss 1968) but the described interspecific differences were minute and based on the size and distribution of KCTs. We examined eggs of several species using the light microscope and found that eggs from a single female can vary, particularly in the distribution of the KCTs. In order to determine if there are consistent interspecific differences in egg structure, further studies utilizing scanning electron microscopy must be performed.

### Remarks

*Heptagenia dolosa, H. townesi*, and *H. patoka* are not known in the larval stage. Based on the female adult and the male subimago, however, *H. whitingi* is easily differentiated from *H. townesi* and *H. dolosa* by the lack of distinct longitudinal dark markings on the abdominal terga. *Heptagenia patoka* is only definitively known from the holotype male imago. We examined the holotype (Illinois Natural History Museum, specimen #16200) and the abdominal coloration is similar to that of *H. whitingi*, but the slide upon which the penes are mounted is missing. Based on the descriptions and figures presented by Burks (1946, 1953), however, there do not appear to be any dorsolateral spines or medial protrusions; both are present on the penes of the subimago male of *H. whitingi*. It is possible that *H. patoka* is actually equivalent to *Raptoheptagenia cruentata* (Walsh) but this cannot be determined until the penes of the holotype are found. Specimens of *H. patoka* have also been reported from Indiana (Randolph and McCafferty 1998) but they were collected from locations where *R. cruentata* has also been collected and thus their identity is uncertain.

**Figure 10.**
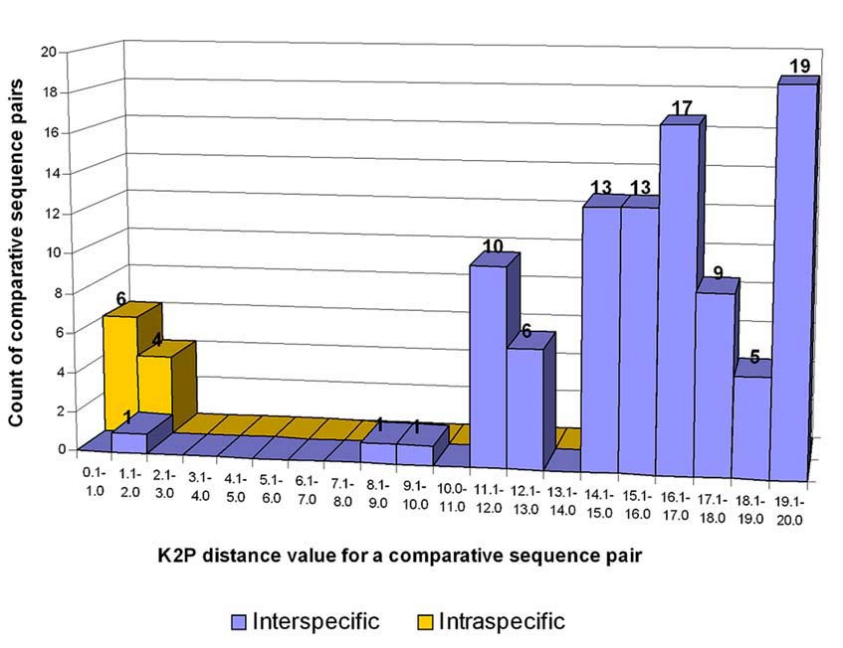
Histogram showing the number of intra- and interspecific pairwise comparisons as a function of K2P distance.

**Table 2.**
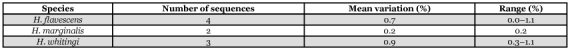
Mean values and ranges of intraspecific sequence divergences (% difference, K2P distance) of COI MtDNA partial sequences of three North American species of *Heptagenia* for which more than one sequence was obtained.

*Heptagenia whitingi* appears to be restricted to large, warm, turbid rivers. In the North and South Saskatchewan Rivers, the new species was only collected over a gravel substrate in fast water. This type of river supports a large number of specialized and rarely collected species such as *Analetris eximia* Edmunds, *Anepeorus rusticus* McDunnough, *Macdunnoa nipawinia* Lehmkuhl, *Lachlania saskatchewanensis* Ide, and *Choroterpes albiannulata* McDunnough.

**Table 3.**
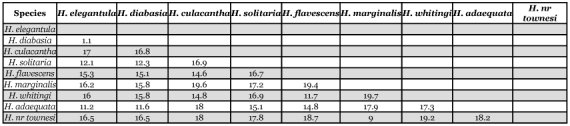
Interspecific divergences (% difference, K2P distance) among COI MtDNA partial sequences of nine species of North American *Heptagenia*. If a comparative pair included at least one species for which multiple sequences were obtained, the mean value of distances between two sequences from the two species in a combination was used.

## Barcode Analysis

### Materials and Methods

#### DNA extraction, amplification and sequencing

13 specimens were sampled representing 8 of the 12 previously named North American species of *Heptagenia*, plus 3 specimens of *H. whitingi* (Table 4). The specimens had been stored in ethanol for periods ranging from less than one year to nine years. A piece of thoracic muscle or a leg from each individual specimen was dissected and rinsed in TE (pH 7.5). A Kontes Grinder was used to grind the tissue in 25 µl of Molecular Grinding Resin (Genotech, gbiosciences.com) in a 1.5 ml microfuge tube. The total genomic DNA was extracted using InstaGene Matrix (Bio-Rad, www.bio-rad.com) according to manufacturer's recommendations. The mixture was incubated overnight at 56 °C, and then boiled and centrifuged as directed by the manufacturer. The supernatant was used as templates for PCR (polymerase chain reaction). The primer pair LC01490 (5′GGTCAACAAATCATAAAGATATTGG 3′ forward) and HC02198 (5′TAAACTTCAGGGTGACCAAAAAATCA 3′ reverse) (Folmer et al. 1994) was employed for the amplification of a 658bp fragment of COI. The temperature and time profile included an initial step of denaturing at 94°C for 1 min, 30 cycles of amplification with denaturation at 94°C for 30 sec, annealing at 43°C for 30 sec, and extension at 72°C for 1 min 30 sec, and a final single extension step, which was carried out at 72°C for 10 min. After being purified, some of the amplified DNA fragments were sequenced by automatic sequencing at the Purdue Genomics Facility, whereas others were cloned into the pGEM-T vector (Promega, www.promega.com) and transformed into *Escherichia coli* strain JM109 (Promega). Plasmid DNA was then extracted using the Qiaprep Spin Miniprep Kit (Qiagen, www.qiagen.com) from bacterial cultures containing inserts of the expected size as determined by PCR. Cycle sequencing of plasmid preparations was carried out using the Big-Dye Terminator v3.1 Cycle Sequencing Kit (PE Applied Biosystems www.appliedbiosystems.com) followed by automatic sequencing at the Purdue Genomics Facility. Both strands of DNA from several clones were sequenced. Sequences for *H. adaequata* and one specimen of *H. whitingi* (reported as *H. flavescens)* were obtained from GenBank (accessions AY3267816 and AY3267915, respectively).

**Table 4.**
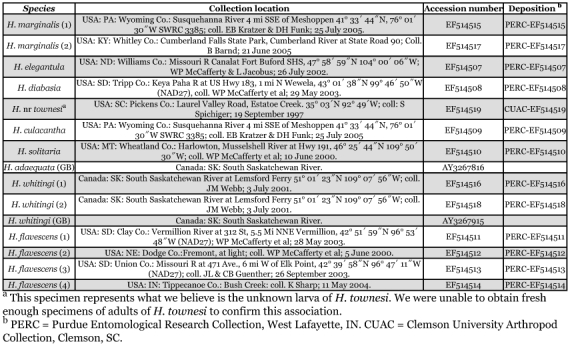
Species identifications, collection locations, GenBank accession numbers, and deposition of specimens analyzed. Sequences obtained from GenBank are indicated with (GB).

**Figure 11.**
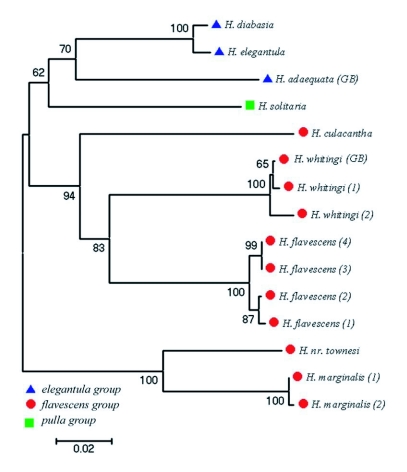
Neighbor Joining tree based on Kimura-2-parameter distances of partial COI mtDNA sequences in MEGA3 for 15 specimens of North American *Heptagenia*. Bootstrap values greater than 50% are shown for respective branches. Species group membership is indicated by symbols located to the left of each specimen.

**Figure 12.**
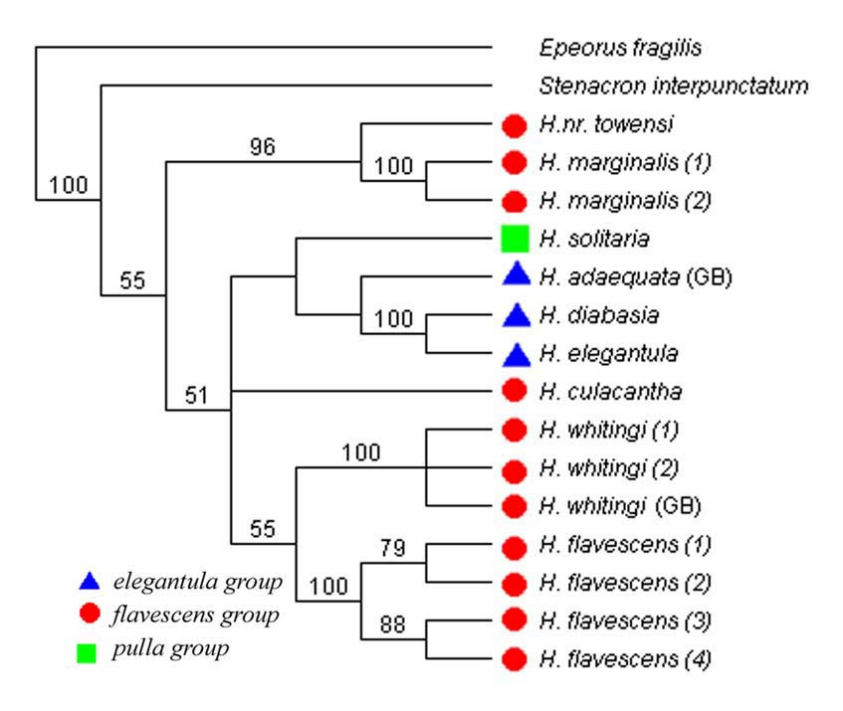
Strict consensus of six Maximum Parsimony trees inferred from partial COI mtDNA sequences in PAUP* for 15 North American *Heptagenia* samples and outgroup specimens of *Epeorus* and *Stenacron*. Bootstrap values greater than 50% are shown for respective branches. Species group membership is indicated by symbols located to the left of each specimen.

Sequences were aligned using ClustalW in MEGA3 (available from www.megasoftware.net) and pruned to 630bp to remove ambiguous base calls. Table 4 shows collection locations and GenBank accession numbers for each specimen.

### Data analysis

A matrix of Kimura-2-parameter (K2P) distances was calculated in MEGA3 (Table 1) and used to construct a neighbor-joining tree. Bootstrap analysis with 1000 replicates was performed on the neighbor-joining tree using MEGA3. The K2P model is appropriate when genetic distances are low (Nei and Kumar 2000) and has previously been used in studies of mtDNA barcoding (e.g., Hebert et al. 2003a, b, Ball et al. 2005, Hajibabaei et al. 2006).

Intraspecific sequence divergences based on K2P distance were calculated as the mean of all intraspecific pairwise divergences for a given taxon. Interspecific sequence divergence based on K2P distance was calculated as the mean of all pairwise divergences between two taxa.

To infer species relationships, a maximum parsimony analysis was conducted in PAUP* (v4.ob10, Sinauer Associates, Sunderland, MA). Sequences of two other Heptageniidae, obtained from GenBank were used as outgroups: *Epeorus fragilis* (Morgan) (GenBank accession AY326821), a member of the Rhithrogeninae, and *Stenacron interpunctatum* (Say) (GenBank accession AY326942), a member of the Heptageniinae. Bootstrap values were calculated with 1000 replicates.

## Results

### Intraspecific sequence divergence

Sequences were obtained from more than one specimen for three of the nine species sampled. Intraspecific sequence divergence ranged from 0.0–1.1% (Tables 1, 2, Figure 10). Mean intraspecific sequence divergence was 0.68%. Intraspecific divergence was ·1.1% for both *H. whitingi* and *H. flavescens*. All of the specimens of *H. whitingi* were from the same locale, whereas each of the *H. flavescens* specimens was from a different location, indicating that sequence divergence was approximately equal within and among populations. Geographic distance was not reflected by the distance data, i.e., *H. flavescens* (3) from South Dakota was more similar to *H. flavescens* (4) from Indiana than it was to either *H. flavescens* (1) or *H. flavescens* (2) from South Dakota and Nebraska, respectively. In every species for which more than one individual was available, the species was monophyletic in every tree.

### Interspecific sequence divergence

Interspecific sequence divergence based on K2P distance ranged from 1.1–20.0% (mean 15.58%) (Tables 1, 3, Figure 10). With the exception of the *H. elegantula -H. diabasia* comparison (divergence = 1.1%), all species-pairs diverged by >8.9% and there was no overlap with intraspecific sequence divergences. The low divergence between *H. elegantula* and *H. diabasia* reflects their dubious specific status (see Discussion). When the *H. elegantula -H. diabasia* pair is excluded from the analysis, mean interspecific sequence divergences ranged from 9.0–19.4% (mean divergence = 15.99%).

### Species relationships

The maximum parsimony analysis returned six most parsimonious trees. The topology of the strict consensus tree of the maximum parsimony results (Figure 12) was largely similar to that of the neighbor-joining tree (Figure 11), the only difference being the uncertain placement of *H. culacantha* in the maximum parsimony analysis. A maximum-likelihood analysis (not shown) was also similar to the neighbor-joining and maximum parsimony results.

The *elegantula* group was found to be monophyletic in both the neighbor-joining and maximum parsimony analyses (Figures 11, 12). The maximum-likelihood results differed in that *H. solitaria* was the sister group of the *H. elgantula + H. diabasia* clade, resulting in a paraphyletic *elegantula* group (not shown). The *flavescens* group was paraphyletic in all analyses. *Heptagenia flavescens* and *H. whitingi* were found to be sister taxa in all analyses, as were *H. marginalis* and *H*. nr *townesi*.

## Discussion

As was suggested for mayflies in general (Ball et al. 2005), we found that sequences of COI successfully distinguished between species of *Heptagenia* because intra- and interspecific sequences differed by at least an order of magnitude in nearly every species comparison. Additionally, every species for which we had more than one specimen was strongly supported as monophyletic. The only species pair that showed lower than expected divergence was *H. elegantula* vs. *H. diabasia*. Morphologically, these species are almost identical with adults differing only by the shape of a marking on the frons, and larvae differing only in the extent of pale spotting. We have found no structural differences in any of the life stages. Differences in genitalia indicated by Burks (1946, 1953) are non-existent. *Heptagenia elegantula* is known as a western species, while *H. diabasia* is known as an eastern species. Their ranges, however, overlap considerably throughout central North America (e.g. McCafferty et al. 2001). Whiting (1985) and Webb (2002) suggested that these two species hybridize in Saskatchewan because larvae and adults with color patterns intermediate between the typical forms for each species were commonly encountered. We have observed similar intermediate forms co-occurring with typical forms of each species in North Dakota, South Dakota and Nebraska. Based on the clinal and inconsistent coloration differences and an absence of morphological differences among populations assigned to these species, together with COI divergence consistent with intraspecific variability, we place *H. diabasia* as a junior subjective synonym of *H. elegantula*, n.syn.

The large gap (more than an order of magnitude) between the intraspecific and interspecific barcode divergence is validation that *H. whitingi* is distinct from *H. flavescens*, with which it has been included in the past (i.e. Lehmkuhl 1976, Whiting and Sheard 1990, Webb 2002, Ball et al. 2005). *Heptagenia flavescens* and *H. whitingi* have slightly overlapping ranges in central North America, but we have not observed any specimens with a color pattern intermediate between the two species, and the difference in the relative size of the spines and setae on the posterior margin of the abdominal terga seems to be consistent as well.

This study provides a preliminary analysis of the relationships among species of North American *Heptagenia*. Although we were unable to obtain usable DNA from *H. Julia, H. patoka, H dolosa, H. pulla* or any positively identified specimen of *H. townesi*, barcode data for at least one species of each species group was analyzed. *Heptagenia julia* and *H. dolosa* are doubtfully good species, however, so their absence would have had a minimal effect on the analysis. The monophyly of the *elegantula* group was supported. Larvae of *H. solitaria* are morphologically more similar to those of the *elegantula* group than they are to those of the pulla-group (unpublished), with which they have traditionally been included. Because we were not able to obtain sequences from either *H. pulla* or *H. julia*, we are unable to determine their relationship to *H. solitaria*. It is not surprising that the *flavescens* group was found to be paraphyletic in all analyses. The character defining the group, the widely divergent penis lobes, is likely plesiomorphic as this form is also found in the closely related genus *Raptoheptagenia* Whiting & Lehmkuhl (Wang and McCafferty 2004).

While a test of barcoding was not an objective of this study, the results conform to the generally observed gap between inter and intraspecific sequence divergence. Using barcodes, it should now be possible to identify previously unidentifiable lifestages of *Heptagenia*, such as subimagos and females. Furthermore, barcodes can be used for the association of larval and adult stages of a species without having to rear specimens.
